# Multimodal gradients of human basal forebrain connectivity

**DOI:** 10.1101/2023.05.26.541324

**Published:** 2023-05-26

**Authors:** Sudesna Chakraborty, Roy A.M. Haast, Prabesh Kanel, Ali R. Khan, Taylor W. Schmitz

**Affiliations:** 1 Neuroscience Graduate Program, Western University; 2 Robarts Research Institute, Western University; 3 Physiology and Pharmacology, Western University; 4 Lawson Health Research Institute, Western University; 5 Institute for Neuroscience, Western University; 6 Medical Biophysics, Western University; 7 CRMBM, CNRS UMR 7339, Aix-Marseille University; 8 Department of Radiology, University of Michigan, MI, Ann Arbor, USA Morris K.; 9 Morris K.Udall Center of Excellence for Parkinson’s Disease Research, University of Michigan, Ann Arbor, MI, USA; 10 Parkinson’s Foundation Research Center of Excellence, University of Michigan, Ann Arbor, MI, USA

**Keywords:** Basal Forebrainn, Connectivity, Human, Neuroimaging

## Abstract

The cholinergic innervation of the cortex originates almost entirely from populations of neurons in the basal forebrain. Structurally, the ascending basal forebrain cholinergic projections are highly branched, with individual cells targeting multiple different cortical regions. However, it is not known whether the structural organization of basal forebrain projections reflects their functional integration with the cortex. We therefore used high resolution 7T diffusion and resting state functional MRI in humans to examine multimodal gradients of forebrain cholinergic connectivity with the neocortex.

Moving from anteromedial to posterolateral BF, structural and functional gradients became progressively detethered, with the most pronounced dissimilarity localized in the nucleus basalis of Meynert (NbM). Structure-function tethering was shaped in part by the distance of cortical parcels from the BF and their myelin content. Functional but not structural connectivity with the BF grew stronger at shorter geodesic distances, with weakly myelinated transmodal cortical areas most strongly expressing this divergence. We then used an in vivo cell type-specific marker of the presynaptic cholinergic nerve terminals, [^18^F] FEOBV PET, to demonstrate that the transmodal cortical areas exhibiting highest structure-function detethering with BF gradients are also among the most densely innervated by its cholinergic projections.

Altogether, multimodal gradients of basal forebrain connectivity reveal inhomogeneity in structure-function tethering which becomes most pronounced in the transition from anteromedial to posterolateral BF. Cortical cholinergic projections emanating from the NbM in particular may exhibit a broad repertoire of connections with key transmodal cortical areas associated with the ventral attention network.

## Introduction

**Figure 1.**
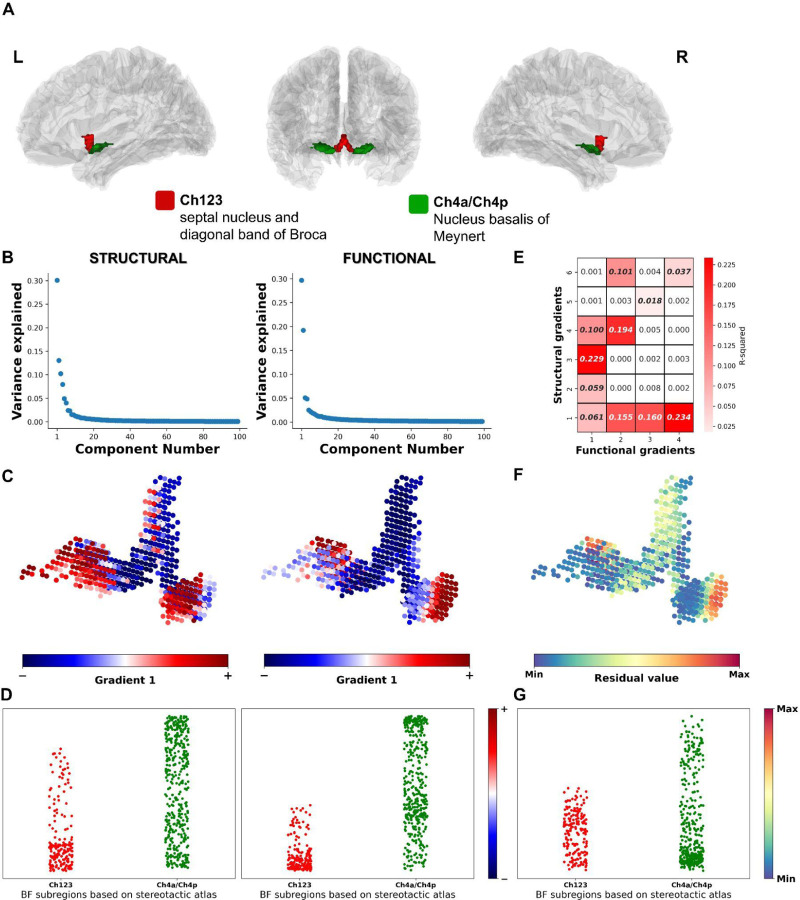
Structural and functional gradients across BF. (A) A 3D view of histologically defined BF subdivisions defined in ref (32) projected on glass brain. (B) Scree plots showing the variance explained by each component of the gradients in structural (left) and functional connectivity (right). (C) The first principal gradient of the BF based on structural (sG1; left) and functional (fG1; right) connectivity both revealed an anteromedial to posterolateral axis. Lower bound of gradient values are represented by blue (and −) while the upper bound is represented by red (and +). (D) Strip plots showing the distribution of BF structural (sG1; left) and functional (fG1; right) gradient distribution within the C123 and C4a/Ch4p stereotactic BF subregions (32). (E) Pairwise R^2^ heatmap of the structural (6 components) and functional (4 components) BF gradients, significant pairs are bolded (p<0.002). (F) Weighted residual values for each of the BF voxels were reconstructed from calculating all the pairwise correlation between structural and functional BF gradients (see Methods). Maximum values (red) indicate divergence between structural and functional BF connectivity. (G) Strip plot showing the distribution of the weighted residuals within the C123 and C4a/Ch4p stereotactic BF subregions (32).

The basal forebrain (BF) (Fig. 1A) is a collection of subcortical cholinergic cell groups which provide the major sources of acetylcholine to the neocortex and hippocampus (1). Structurally, the ascending cholinergic projections are highly branched, with individual cells often targeting multiple different cortical areas (2–4). The total arborization of a single human cholinergic BF neuron is estimated to have a length in excess of 100 meters (4).

The organization of ascending BF cholinergic projections may reflect complex spatial topographies of connectivity with the cortex (5, 6). Within the BF, subregional structural changes in gray matter and white matter integrity are associated with distinct patterns of cortical degeneration and cognitive dysfunction (7–11). In neurodegenerative diseases such as Alzheimer’s (AD), early dysfunction or loss of specific BF cholinergic fibers may alter local neuronal functions in cholinoreceptive cortical areas (12). Consistent with a topographical organization, axonal tracing studies suggest that BF cholinergic neurons are grouped into ensembles which target functionally interrelated cortical areas (13). Moreover, patterns of functional connectivity in distinct BF subregions have been found to overlap with distinct cortico-cortical networks (14–16). Although these separate lines of evidence suggest the cortex expresses topographies of BF structural and functional connectivity, the intermodal relationship of these topographies to one another is unknown.

How does the structural organization of cholinergic BF projections relate to their functional integration in the cortex? One possibility is that BF structural and functional connectivity is closely tethered. In tethered connections, spatially varying profiles of white matter projections and hemodynamic co-fluctuations within the BF would overlap to one another and share common cortical targets. Studies examining profiles of cortico-cortical white matter and resting state connectivity consistently observe strong intermodal tethering in unimodal cortex, which is thought to reflect a preponderance of highly myelinated short range connections among neuronal populations with similar functional repertoires (17, 18). Alternatively, BF structural and functional connectivity may diverge from one another, exhibiting little overlap within the BF and distinct cortical fingerprints. In cortico-cortical connectivity, this profile of structure-function detethering is observed in the association cortex, where weakly myelinated longer range connections provide neuronal populations with greater integration and a more diverse functional repertoire (17, 18).

Here we addressed the relationship between structural and functional connectivity in the ascending basal forebrain projections. We used multimodal imaging combining high resolution 7 Tesla (7T) diffusion (dMRI) and resting-state functional MRI (rsfMRI) in a cohort of 173 individuals from the Human Connectome project (19). We derived gradients of the BF in each modality (20) employing diffusion map embedding to elucidate fine-grained continuous maps of its connectivity. To quantify intermodal tethering, we computed the residual variance between (a) the gradients of structural and functional connectivity within the BF and (b) the expression of these gradients on the cortical surface. This method allowed us to ask if spatial topographies of BF structure and function overlap one another and whether the degree of this spatial overlap is homogeneous or inhomogeneous across different BF subregions. We found greater inhomogeneity in structure-function tethering moving from anteromedial to posterolateral BF, with the strongest inhomogeneities localized in the nucleus basalis of Meynert (NbM).

Next, we explored the spatial overlap of the structural and functional gradients with known cortico-cortical networks, focusing on the most dominant BF gradient observed in each imaging modality and computing their gradient-weighted cortical maps. Similar to the BF gradients, we quantified the similarity between the structural and functional gradient-weighted cortical maps by calculating their residuals. The resulting residual cortical map exhibited pronounced dissimilarity in midcingulo-insular cortical areas associated with the ventral attention network (21–29). Finally, we examined what may account for this structure-function detethering by examining its spatial relationships to: (a) the cortical geodesic distances from BF, (b) cortical myelination estimated from the T1w/T2w ratio (30), and (c) the cortical concentrations of cholinergic nerve terminals estimated from cell type specific molecular imaging of the presynaptic vesicular acetylcholine transporter (VAChT). We found that the higher BF structure-function detethering corresponds to shorter distance from the BF, lower myelination and relatively higher concentrations of cholinergic innervation.

## Results

We used high-resolution 7T MRI HCP data (n=173) (31) and a widely used stereotactic atlas of the BF (32) to build structural (dMRI) and functional (rsfMRI) connectomes. Because the BF is an anatomically small subcortical structure, we also validated the BF tracts identified by dMRI against prior BF tractography findings from *ex vivo* tracing (33) and *in vivo* dMRI (10, 34) in humans (Supplemental Fig. S1). Consistent with those studies, the *in vivo* tractography results from our study reveal the strongest weighting in two white matter pathways emanating from the BF, a medial cingulum pathway and lateral capsular/perisylvian pathway. Individual connectomes were averaged and reduced to a 2-dimensional *m*-by-*n* matrix, where *m* represent the voxels in the BF ROI and *n* are the cortical targets (35) with their corresponding connectivity strengths. To capture the gradients, we used diffusion embedding - a nonlinear dimension reduction approach that identifies multiple axes of variation in connectivity along the BF voxels - separately for structural and functional connectivity matrices (20).

### Primary structural and functional BF gradients

The first gradient for both structural and functional connectivity data explained the most (30%) variance (Fig. 1B) followed by a reduction in explanatory power by 50% (~15%) for the second gradient. Given this dominance of the first gradients, we therefore focused on the first, principal gradient in each modality. In both the structural and functional gradient, a smooth gradient transition from anteromedial to posterolateral BF was observed (Fig. 1C). This gradient broadly recapitulates prior anatomical and electrophysiological work in mouse, rat and macaque (36–44) which distinguishes connectivity profiles, neuronal firing patterns and neurodevelopmental trajectories between anteromedial and posterolateral cholinergic nuclei of the BF.

We then examined if this anteromedial-to-posterolateral gradient patterns differentiated known histologically defined subregions of the BF (Fig. 1A), namely the Ch123 subregion containing the septal nucleus and diagonal band of Broca (in red), versus the Ch4a/Ch4p subregion containing the NbM (in green). To do so, we used permutation tests with fitted surrogate maps (see Methods) to test if the distributions of gradient values within Ch123 and Ch4a/Ch4p differed from one another. We performed tests to compute the difference in both the means and coefficients of variation (CoV) between Ch123 and Ch4a/Ch4p. The CoV is a statistical measure used to express the degree of variation of a set of data relative to its mean. It is defined as the ratio of the standard deviation to the mean of the data, expressed as a percentage. A higher CoV indicates a greater degree of variability or dispersion in the data, while a lower CoV indicates less variability or greater consistency. The mean gradient values were significantly different between Ch123 and Ch4a/Ch4p for structural (mean difference=−0.336, p_perm_=0.05) and functional (mean difference=−0.469, p_perm_=0.003) connectivities. The Ch123 region exhibited higher overlap with the gradient lower bound (blue) whereas the Ch4a/Ch4p exhibited higher overlap with the gradient upper bound (red) (Figure 1D). We also observed that CoV was significantly different between Ch123 and Ch4a/Ch4p, indicating that gradient values in Ch123 exhibit greater dispersion than Ch4a/Ch4p for both sG1 and fG1 (p_perm_<0.001). This latter effect is due to the skew of the distribution in Ch123 relative to the mean.

### Multimodal gradients of BF structure-function relationship

**Figure 2.**
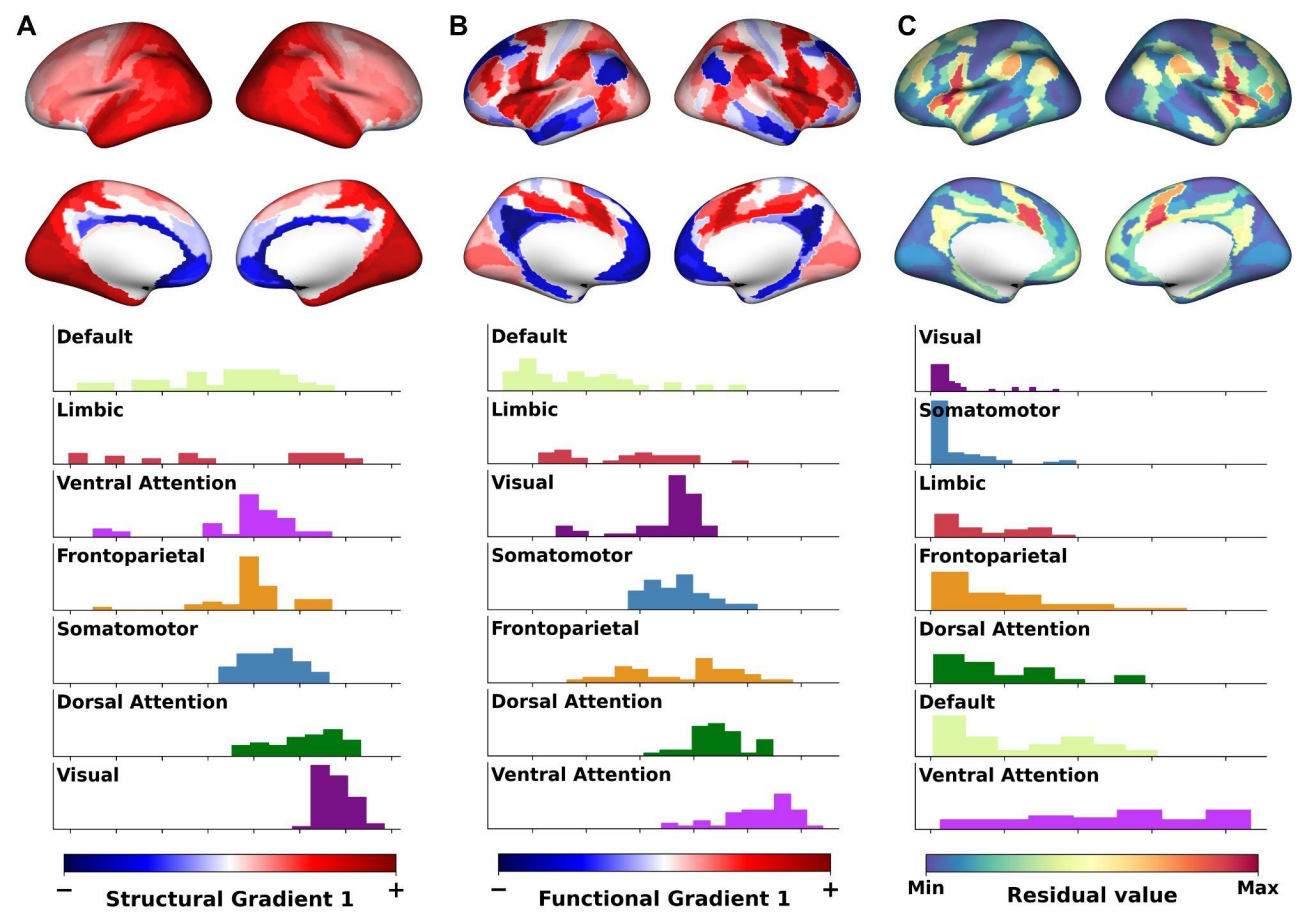
Structural and functional gradient-weighted cortical maps and their relationship. (A) Structural G1-weighted map projected to the cortical surface (top), the black dot represent the BF seed, lower bound of G1-weighted gradient values are represented by blue (and −) while the upper bound is represented by red (and +); and histogram plot (bottom) showing the distribution of G1-weighted gradient values separately for each of the 7 networks color-coded based on the ref (28) (Supplemental Fig. S3). The networks are ordered by the mean values. (B) Functional G1-weighted cortical map (top) and histogram plot (bottom) of network distribution ordered by the mean values. (C) Weighted cortical residual map calculated similarly to weighted residual map of BF (Fig. 1F; see Methods) and the corresponding distribution of the residual values for each of the 7 networks ordered by their mean values.

We next examined the magnitude of shared variance, or tethering, between BF structural and functional gradients to determine their spatial similarity to one another. Although sG1 and fG1 represent the most explanatory intra-modal gradients, they are not necessarily the most explanatory pair of gradients in terms of shared intermodal variance. It could be the case, for example, that sG1 and fG2 share more spatial similarity than sG1 and fG1. To explore this possibility, we computed the voxelwise intermodal associations (R^2^ value) for all structural and functional gradients whose initial components fell above the variance plateau (Fig. 1B). This yielded a matrix of 24 structure-function gradient pairs (sG1-6 and fG1-4). Figure 2E shows the heatmap matrix of R^2^ values for each pairwise structure-function gradient (significant ones are bolded p<0.002). The shared variance across all 24 pairs was low (mean R^2^=0.05). However, the range of R^2^ varied from 1.4 × 10^−4^ to 0.23.

We then computed voxelwise regressions for each of the 24 structure-function gradient pairs in this matrix and extracted their corresponding residuals, which quantify the magnitude of unexplained variance for that pair. All 24 pairs of residuals were weighted according to the initial variance explained (Fig. 1B) for that pair and then summed to produce a weighted average residual map encoding BF structure-function detethering (see Methods). This weighted average residual map was projected back to BF voxel space, which revealed an anteromedial to posterolateral topography, with the highest structure-function detethering localized in posterolateral subregions (Fig. 1F). Examining this detethering pattern according to the histologically defined boundaries of Ch123 and Ch4a/4p(32), the mean weighted residual values were not different between the two subregions (mean difference=−0.008, p_perm_=0.40) (Figure 1G). However, the residual values in Ch4a/Ch4p exhibited significantly greater variability in comparison to Ch123 (CoV difference=−34.78, p_perm_=0.01). This latter finding suggests that structure-function detethering within Ch4a/Ch4p was more inhomogeneous than Ch123.

### The cortical expression of BF structural and functional gradients

We next computed gradient-weighted cortical maps (45) to determine how BF gradients were expressed by the cortex. The gradient-weighted cortical maps were created by multiplying each row of the initial connectivity matrix (*M*_BF voxels_ x *N*_cortical parcels_) with the corresponding sG1 or fG1 value to create a gradient-weighted connectivity matrix (*GM*_BF voxels_ x *N*_Cortical parcels_). Finally, all rows of this gradient-weighted matrix (i.e. *GM*_BF voxels_) were averaged to produce a single cortical representation of the particular gradient (see Supplemental Fig. S2 and Methods).

For the gradient-weighted cortical map corresponding to sG1 (sG1ctx; Fig. 2A top), we observed a smooth macroscale transition from the anteromedial to posterolateral cortical surface. By contrast, the gradient-weighted cortical map corresponding to fG1 (fG1ctx; Fig. 2B top) exhibited a more patch-like pattern. We then examined if the spatial topographies of sG1ctx and fG1ctx exhibited any relationship to the spatial topographies of intrinsic cortico-cortical resting state networks (28). To do so, we examined the distributions of gradient values captured by each of 7 macroscale resting state networks covering the entire human cerebral cortex (Fig. 2AB bottom).

A pattern in which the distributions of these gradient values are well delineated from one another across different networks would be consistent with a high level of topographic mapping between specific networks and specific spatial locations along the anteromedial to posterolateral axis of the BF. A pattern in which the distributions of weights are more spread out and overlapped across different networks would be consistent with low topographic mapping. For both sG1ctx and fG1ctx, we observed an intermediate pattern of delineation, which was stronger for some networks than others. For example, we observed greater spread across the default mode, limbic, ventral attention and frontoparietal networks. A common feature of these networks is that they have hubs located exclusively in the transmodal cortex (see supplemental Fig. S3 for the distribution of the 7 networks on the cortical surface).

As done for the BF gradients, we quantified the similarity between the structural and functional gradient-weighted cortical maps by calculating their pairwise unexplained variance (i.e., residuals) across cortical parcels. The resulting residual cortical map (Fig. 2C top) exhibited increasing dissimilarity moving from unimodal to transmodal cortex, with highest dissimilarity in the anterior cingulate cortex. These cortical parcels with greater structure-function dissimilarity tended to overlap primarily with the ventral attention network (Fig. 2C bottom).

### BF structure-function tethering is shaped by cortical geodesic distance and myelination

What could be the reason behind the observed structure-function detethering in the transmodal cortex? Structure-function tethering accounts of cortico-cortical connectivity propose that the divergence between a cortical area’s functional and structural connectivity increases as a function of its geodesic distance from unimodal sensory cortex (17, 46, 47). This is due to cortical expansion, which disproportionately affects the more recently evolved association cortices. Under this account, phylogenetically newer cortical areas are less constrained by the short-range wiring of sensory cortex, yielding higher levels of functional integration and divergence from structural connectivity, particularly among areas in the frontoparietal cortex. One possibility is that, like increasing geodesic distance from the unimodal cortex, increasing geodesic distance from BF would be associated with increased detethering of structure and function. However, our residualized BF gradient weighted cortical maps suggest a striking inversion of this pattern (Fig. 2C top). At closer rather than farther distances from the BF, structure and function were more detethered (more unexplained variance between modalities), with the most unexplained variance in proximal hubs of the ventral attention network. By contrast, the least unexplained variance was concentrated in distal visual and somatomotor networks.

**Figure 3.**
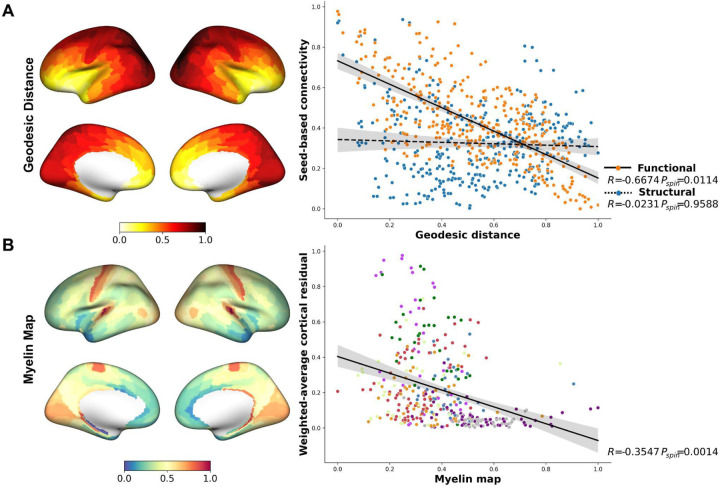
Multimodal connectivity in relation to cortical geodesic distance and myelination. (A) Parcellated (35) geodesic distance (left) from the cortical BF label (black spot) to each point on the cortical surface, darker red indicating farther geodesic distance from the BF seed and scatter plot (right) of the structural (blue) and functional (orange) seed-based connectivity against the geodesic distance demonstrating significant negative correlation for the functional connectivity but no relationship with structural connectivity. Each point in the scatter plot represents cortical parcels based on Glasser parcellation (35) and spin test using spatial null model (48) results are reported in the box corresponding to the scatter plots. (B) Parcellated T1w/T2w ratio (myelin) map provided by the HCP (30) and averaged across subjects with the cortical BF label indicated by black dot, red color indicating stronger myelination while blue indicates weaker (left); and scatter plot of weighted cortical residual map against the myelin map showing significant negative relationship (right). Each point in the scatter plot represents cortical parcels and is color-coded by the 7 network (28) identical to Supplemental Fig. S3.

To more directly explore constraints of distance on BF-cortical connectivity, we examined our original structural (number of white matter streamlines) and functional (Pearson *r*, encoding hemodynamic correlations) BF seed-based connectivity maps in relation to the intrinsic geometry of the cortex measured by geodesic distance from BF to each cortical parcel. We started with creating an approximate BF seed label on the cortical surface. This is done by sampling the original volumetric BF mask onto the subject’s white matter (WM) surface across all subjects, average them to get a probability map and then thresholded for a final binary BF label on the cortical surface (see Methods). This BF seed was then used to calculate the minimum geodesic distance between all points on the cortical surface and the seed. Finally, since our seed-based connectivity maps are parcellated based on the HCP-MMP 1.0 (35) this geodesic distance map was parcellated using the same surface atlas which is visualized on the inflated cortical surface (Fig. 3A left). We then quantified the spatial relationships of the geodesic distance map with each modality-specific connectivity map using spin tests against spatial null models (48, 49). We found that the relationship of BF-cortical structural connectivity with BF-cortical geodesic distances was negligible (R=−0.02, p_spin_=0.96). By contrast, a significant negative correlation was detected between BF-cortical functional connectivity and BF-cortical geodesic distances (R=−0.67, p_spin_=0.01). The magnitude of this association was significantly higher than that observed for structural connectivity (average difference of −0.624 between correlation coefficients with 95% CI [−0.51,−0.73], p_boot_<0.001, as revealed by bootstrap analysis). The strength of BF connectivity diverged more strongly between modalities in transmodal cortical areas at smaller geodesic distances from BF (Fig. 3A right). Hence, these findings provide quantitative support for our observation that structure-function detethering tends to increase in cortical areas at decreasing geodesic distances from BF.

A second and related explanation given for structure-function detethering comes from work examining the relationships of rsfMRI measures of cortico-cortical connectivity with cortical myelin content (17, 50). From this work, close tethering between structural and functional connectivity is consistently observed among areas in unimodal cortex, where temporal co-fluctuations in hemodynamic responses coincide with highly myelinated short-range white matter connections. Transitioning to association cortices, function detethers from structure as hemodynamic co-fluctuations among different cortical areas increasingly reflect weakly myelinated long-range connections. We therefore examined if structure-function detethering of BF gradient weighted cortical maps also reflected cortical myelin content. To do so, we examined the spatial relationship between our gradient-weighted cortical residuals map (Fig. 2C top) and a group averaged map of cortical myelin content (35) using spin tests (48, 49). Individual myelin maps provided by the HCP (30) were averaged across subjects to create a group myelin map. This group myelin map was then transformed to the *10k_fsavg* surface space and parcellated using the Glasser atlas (35) and projected on the inflated surface (Fig. 3B left). Consistent with patterns observed for cortico-cortical connectivity, we found that the highest magnitudes of BF detethering were localized to the most weakly myelinated areas of transmodal cortex (R=−0.355, p_spin_=0.001; Fig. 3B right).

### BF structure-function tethering reflects the density of cortical cholinergic innervation

Why might structure and function diverge in weakly myelinated cortical regions situated at closer distances to BF? Due to its anteromedial location in the brain, the BF is closer to many transmodal cortical areas than the unimodal cortex. However, this proximity to transmodal cortical areas does not explain why BF structure and function would exhibit closer tethering in cortical areas at greater geodesic distances. One possibility is that the number of axon terminals (branches) per cholinergic neuron varies from cell to cell in the BF. Under this account, cortical areas expressing divergent structure-function tethering with BF may receive more inputs per cholinergic neuron (cells with more branches), while cortical areas expressing closer structure-function tethering with BF may receive fewer inputs per cholinergic neuron (cells with fewer branches).

**Figure 4.**
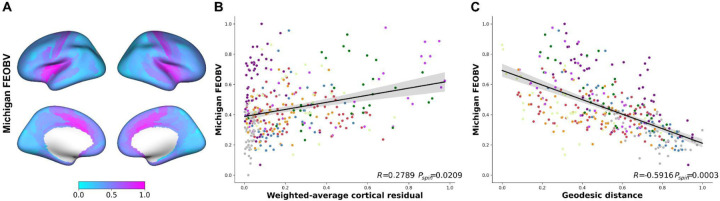
Cortical cholinergic innervation in relation to cortical residual map and geodesic distance. (A) Parcellated and rescaled FEOBV PET map, pink indicating higher values while sky blue color indicating lower with the cortical BF label indicated by black spot. (B) Scatter plot against the weighted cortical residual map indicating positive correlation. Each point in the scatter plot represents cortical parcels based on HCP-MMP 1.0 parcellation (35) and is color-coded by the 7 network (28) identical to Supplemental Fig. S3. Spin test using spatial null model (48) results are reported in the box corresponding to the scatter plots. (C) Scatter plot of the FEOBV PET map against the geodesic distance showing significant negative relationship.

We tested this hypothesis using in vivo positron emission tomography in combination with the [^18^F] FEOBV (51, 52), a radiotracer which binds to the vesicular acetylcholine transporter (VAChT). The VAChT is a glycoprotein expressed solely by cholinergic neurons, with the highest density of binding sites on the presynaptic terminals. We acquired intensity normalized distribution maps of [^18^F]FEOBV binding from a group of healthy cognitively normal young adults (N=13; mean age=24.54, 3 females) (53), and produced an average map representing the BF cortical cholinergic projectome (Fig. 4A; see Methods).

Using spin tests against spatial null models (48, 49), we extracted the cortical expression of BF structure-function detethering (cortical residualized map from Fig. 2C top) and cholinergic innervation estimated from VAChT concentrations using the common cortical parcellation (35). Consistent with our hypothesis that BF neurons are diverse in terms of branch complexity, we found that cortical areas exhibiting greater divergence in BF structure-function tethering also exhibit greater density of BF cholinergic input, i.e. higher VAChT concentration (R=0.28, p_spin_=0.02; Fig. 4B). Geodesic distances from the BF to cortex also reflected the spatial distribution of the cortical cholinergic innervation: cortical areas closer to the BF tended to express higher VAChT concentrations (R=−0.592, p_spin_=0.0003; Fig. 4C). We replicated these associations with three other atlases of FEOBV PET publicly available (49) and found similar results of showing significant (p_spin_<0.05) positive spatial correlation with cortical residual maps for all three VAChT maps and significant negative spatial correlation with geodesic distance in two of the maps (Supplemental Fig. S4).

**Figure 5.**
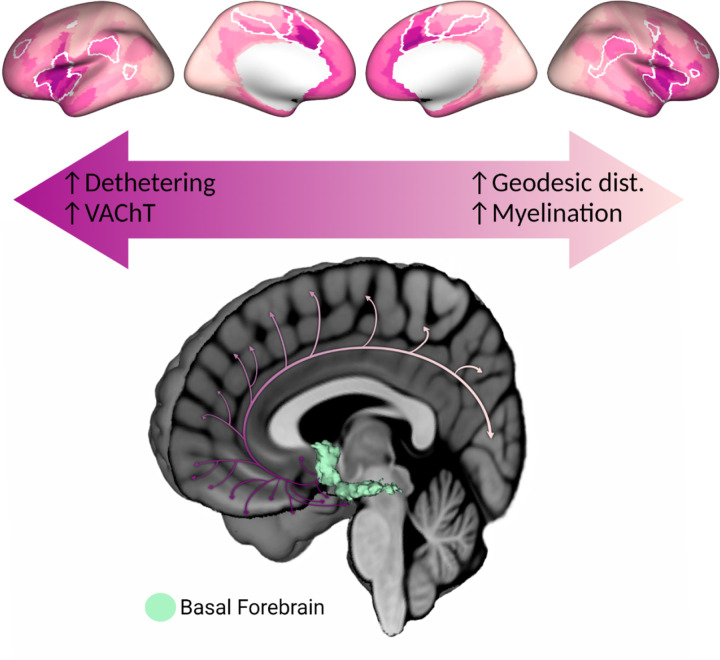
Comprehensive average of surface maps highlighting multimodal features of the BF cholinergic projectome. (Top) The following maps were rescaled to a common range and averaged together: (1) The cortical map encoding structure-function residuals in BF connectivity (detethering; Fig. 2C top), (2) the FEOBV PET map (VAChT; Fig. 4A), (3) the BF geodesic distance map (Fig. 3A left) and (4) the myelin map (T1w/T2w ratio; Fig. 3B left). For the geodesic distance and myelin maps, values were inverted prior to averaging such that darker pink areas reflect higher structure-function detethering, higher VAChT concentration, shorter BF geodesic distances and weaker myelination. The BF seed label is indicated by the black dot and the ventral attention network (28) is outlined with white solid line. (Bottom) Graphical schematic summarizing our findings. A 3D render of the BF probabilistic atlas (32) is superimposed on a mid-sagittal cross-section of the MNI152 template brain. The cortical cholinergic projections were created with BioRender.com. Darker pink arrows indicate higher detethering and VAChT concentration, shorter geodesic distance and weaker myelination while the faded color indicates lower detethering and VAChT concentration, longer geodesic distance and stronger myelination.

Altogether our findings reveal a multimodal gradient in the human cerebral cortex which expresses both BF connectivity and cholinergic innervation. Along this gradient, cortical areas which express divergent BF structure-function coupling, shorter distances from the BF and weaker myelination receive dense cholinergic innervation from highly branched neurons (Fig. 5). These areas closely resemble hubs of the midcingulo-insular network (24), which comprises hubs of the ventral attention and salience networks (21–23, 25–28). Hubs of the dorsal attention and default mode networks express an intermediate profile in this gradient. By contrast, cortical areas which express convergent BF structure-function coupling, larger distances from the BF and stronger myelination receive sparse cholinergic innervation from neurons with fewer branches. These areas tend to overlap the primary visual and sensorimotor cortex, suggestive of a hierarchical organization in BF cholinergic innervation.

## Discussion

Extending on prior research in mouse, rat and macaque, we provide evidence in humans that the BF exhibits an anteromedial to posterolateral gradient of structural and functional connectivity with the cortex. Although the axes of these multimodal gradients are qualitatively similar to one another, quantitative comparison of their spatial organization revealed localized structure-function detethering, with the strongest detethering concentrated in the posterolateral NbM. Examination of where structure-function detethering was most strongly expressed in the cortex revealed a set of midcingulo-insular hubs within the ventral attention network. Further analyses of cortical properties thought to shape structure-function tethering, including interregional geodesic distances and myelin content, revealed novel insights into how BF-cortical connectivity differs from cortico-cortical connectivity. We found that structural and functional connectivity is more divergent in cortical areas at closer, as opposed to farther, geodesic distances from the BF. These proximal cortical areas tend to be weakly myelinated. To determine what features of BF connectivity may account for this unexpected pattern, we combined our multimodal MRI analyses of BF connectivity with *in vivo* PET imaging of VAChT, a cell type specific marker of the presynaptic BF cholinergic cortical projectome. We found that cortical areas with higher VAChT tend to express greater BF structure-function detethering, consistent with a highly branched innervation whereby individual BF cholinergic neurons target diverse cortical areas with numerous axonal collaterals.

Cell type specific labeling work in non-human animal models indicates that the axonal projections of BF cholinergic neurons vary in terms of their branch complexity, both in terms of total number of branches per cell and diversity of cortical targets (2–4, 54, 55). Collectively, our findings in humans indicate that this diversity in branch complexity is reflected by BF structure-function tethering(5). Under this framework, cortical areas expressing higher structure-function detethering receive input from BF cholinergic neurons with higher branch complexity. The populations of BF cholinergic neurons providing these highly branched projections tend to be located in the posterolateral NbM, where structure-function gradient divergences were higher and more variable than the medial septum and diagonal band of Broca (Fig. 1FG). Our *in vivo* findings are consistent with postmortem histology evidence in humans, which indicate that cholinergic neurons in the NbM have larger arborizations than those in anteromedial nuclei (56).

In rodents, the medial septum and diagonal band are as large as the NbM. In macaques and humans, by contrast, the NbM is considerably larger than medial septum and diagonal band (43). The human NbM is also more densely populated with choline-o-acetyltransferase (ChAT) expressing cholinergic neurons (~90% of cells) compared to medial septum (10%) and diagonal band (max ~70%) (1, 57, 58). The phylogenetic structural progression of the NbM’s size and complexity may reflect the evolutionary expansion of the transmodal cortical areas it projects to (43, 56, 59). Nevertheless, the human NbM is estimated to contain only about 400,000 cholinergic neurons (60), a small proportion of the ~16 billion neurons estimated for the human cerebral cortex (61). If every neuron in the cerebral cortex directly synapsed with an NbM cholinergic fiber, this would require ~40,000 branches per axon. A more likely scenario is that individual NbM cholinergic fibers provide input at the level of cortical ensembles, i.e., groups of neurons with similar feature tuning. Although the size of these ensembles varies across cortical areas, *in vivo* (62) and *in silico* (63) evidence suggests that the upper bound for ensemble size is ~200 neurons. Assuming a uniform average ensemble size of 100 neurons, the number cortical targets decreases to 160,000,000. In this architecture, the ratio of cortical ensembles to NbM cholinergic neurons is 160,000,000:400,000, which necessitates only ~400 branches per cholinergic neuron to provide complete innervation. This latter estimate is well within the range of empirically verified axonal branch counts for individual BF cholinergic neurons (4).

How might the observed gradient of cortical cholinergic innervation translate to the role of acetylcholine signaling in attention? When ensembles of neurons receive driving input from their preferred stimulus features, the responses of individual neurons are suppressed, or normalized, by the total activity of its ensemble and neighboring ensembles (64). This divisive normalization moderates noisy responses from individual neurons and prevents runaway excitation. Directed attention is thought to bias these mutually suppressive competitive interactions among ensembles, enabling some stimulus representations to dominate over others (65, 66). Spatially localized acetylcholine release at the level of cortical ensembles may represent a key neurochemical basis of these biasing signals (67). However, it remains poorly understood whether and how acetylcholine signaling changes from unimodal to transmodal stages of the cortical hierarchy. Our findings imply that the branch complexity of cholinergic projections may reflect properties of the cortical ensembles they target. Moving up the cortical hierarchy, neuronal ensembles with increasingly diverse repertoires or cortical-cortical connectivity may similarly receive input from increasingly branched BF cholinergic neurons. In terms of hierarchical integration, the midcingulo-insular hubs of the ventral attention network may represent the apex of the BF cortical cholinergic innervation. This proposal is in line with human connectomic research indicating that the midcingulo-insular network plays a supervisory role in directing attentional biasing signals throughout the brain (21–29).

A diversity of branch complexity in BF cholinergic neurons may also account for differences in their vulnerability to aging and disease. Cell type specific labeling and transcriptomic analyses examining morphological and functional properties which increase a neuron’s vulnerability to age-related neurodegenerative disease such as AD have consistently demonstrated large axonal projections as a key risk factor (4, 68–70). The observed structure-function detethering in the BF, and in particular the NbM, is consistent with neurons exhibiting large arborizations. This morphofunctional property of NbM cholinergic neurons may increase their vulnerability to dysfunction in the aging brain. In parallel, our observation that ventral attention network may receive input from the most highly branched NbM cholinergic neurons implies that these cortical areas might exhibit higher vulnerability to dysfunctional cholinergic signaling in the aging brain. It is also notable that the BF cholinergic neurons with fewer arborizations, which our findings suggest primarily target the primary and somatosensory cortices, constitute projection zones which are relatively spared by pathology in early stages of AD (71).

Our findings are subject to several important methodological considerations. First, the basal forebrain is a small subcortical structure with poorly defined anatomical boundaries. We therefore used a probabilistic atlas to localize its constituent nuclei. However, when using probabilistic BF atlases in combination with data collected at spatial resolutions typical of 3T structural (1.5 mm^3^) and functional MRI (3 mm^3^), aliasing of adjacent structures has been shown to systematically overestimate the BF gray matter (72). To mitigate this issue, we used high spatial resolution dMRI (1.05 mm^3^) and rsfMRI (1.6 mm^3^) data acquired at 7T. Second, our measures of structural connectivity were estimated using streamline tractography on diffusion-weighted imaging, which can be susceptible to false positives and negatives in certain brain areas (72). It is therefore possible that the regional variation in BF structure–function correspondence is partly explained by regional variation in tractography performance. Another concern is the susceptibility-related spatial distortions near the BF region for fast readout scans, such as used for the rsfMRI acquisitions by the HCP. Although corrected for using a separately acquired field map, these spatial distortions might lead to suboptimal probing of BF voxels in such data with possible contamination from white matter (WM) tissue and cerebro-spinal fluids (CSF). To limit the impact of the latter on the functional timeseries analysis, additional denoising using the average WM and CSF timeseries was performed.

In sum, we demonstrate that multimodal gradients of BF connectivity reveal spatially inhomogeneous patterns of structure-function tethering in the cortex, with the lowest tethering in mid-cingulate and anterior insular cortical areas involved in salience detection and allocation of attentional resources throughout the brain. These cortical areas tend to be located proximal to the BF, receive disproportionately higher concentrations of cholinergic innervation, and exhibit lower myelination.

## Materials and Methods

We used high-resolution minimally pre-processed 7T MRI HCP data (n=173) (31) and the existing stereotactic atlas of the BF (32) to build structural and functional connectomes. Any further pre-processing and connectivity matrix construction was done on the compute cluster. Workflows were built using Snakemake (73) with the full workflow available on GitHub (see data and code availability for specifics). Individual connectomes were averaged and reduced to a 2-dimensional *m*-by-*n* matrix describing the pairwise connectivity strength between *m* BF ROI voxels and *n* cortical regions (35). The BrainSpace toolbox (20) was used to capture the gradients which, as well as any further analysis, was done using Jupyter Notebook (74).

### Data Acquisition

High-resolution 7T dMRI and rsfMRI data were downloaded from the HCP data repository (19). We used the minimally pre-processed data described in ref (31) consisting of 173 healthy subjects (69 male, 104 female) aged 22 to 35 years. The dMRI images were collected with 1.05 mm^3^ isotropic voxels, TR=7000 ms, TE=71.2 ms, b-values=1000, 2000 s/mm^2^, FOV=210 × 210 mm^2^. Resting-state fMRI images were collected with 1.6 mm^3^ isotropic voxel size, TR=1000 ms, TE=22.2 ms, FOV=208 mm^2^, spanning 4 runs of 16-minute duration each, per subject. For anatomical imaging, two T_1_-weighted (T_1_w) scans were obtained using a three-dimension (3D) magnetization-prepared rapid gradient-echo (MPRAGE) (75) sequence and two T_2_-weighted (T_2_w) images using a 3D T_2_-SPACE sequence, all with identical geometries and a 0.7 mm^3^ isotropic voxel size. Full details of the acquisition parameters can be found in the HCP S1200 release reference manual (76).

### Basal Forebrain Mask

The basal forebrain (BF) region-of-interest (ROI) was created using the existing stereotactic atlas of the BF (32). This stereotactic BF atlas is based on histological sections obtained from 10 postmortem brains, the magnocellular cell groups were delineated in each slice, 3D reconstructed and warped into the MNI single-subject reference space (77). The atlas consists of 4 subregions of the BF defined in the nomenclature: Ch1–2, Ch3, Ch4, and Ch4p. For each subregion, a stereotactic probabilistic map has a range of 0 to 10 indicating the number of brains containing the specific magnocellular cell groups in the given voxel. Our BF ROI is created by thresholding these subregion masks to 50% first and then combining all to get a mask covering full BF. This BF ROI mask was then warped into MNI152 non-linear 6^th^ generation atlas (MNI152Nlin6Asym) (77).

### Structural Connectivity Reconstruction

Diffusion tractography was performed to get a connectivity matrix for diffusion data. As part of the minimal preprocessing pipeline data release, all subjects underwent FreeSurfer processing (v5.3.0-HCP) (78). The BF ROI mask was then first resampled and transformed to the individual subjects’ minimally preprocessed volume space (0.7mm^3^). Volumetric cortical labels were built by mapping the HCP-MMP 1.0 surface parcellation (35) using Connectome Workbench’s ribbon-constrained *label-to-volume-mapping* function and FreeSurfer-derived surfaces. The BF ROI voxels were used as seeds, and the 180 cortical regions in each hemisphere were combined and used as targets to perform probabilistic tractography using FSL’s *probtrackx* (79) with 5000 streamlines per BF ROI voxel (see Supplemental Fig. S1). The resulting probability maps in the BF quantified the number of streamlines that reached each target. The maps were resampled to MNI space (80) in 1.6mm^3^ resolution to match the functional connectivity matrix and reduced to a 2-dimensional *m*-by-*n* matrix, where *m* represents the voxels in the BF ROI (599 voxels) and *n* is the cortical targets (180 each hemisphere) with their corresponding number of streamlines. This *m*-by-*n* connectivity feature matrix for all 173 subjects was averaged to calculate the gradients.

### Functional Connectivity Reconstruction

First, the BF ROI mask in the minimally preprocessed volume space was resampled to 1.6mm^3^ isotropic voxel size to match resolution of the rsfMRI data and added to the subject’s subcortical parcellation. A functional connectivity matrix was then created for each subject by calculating the temporal correlation between BF voxels and cortical ROIs. All four runs (i.e., two sets of 16 min. runs with posterior-to-anterior and anterior-to-posterior phase-encoding) of the minimally preprocessed and ICA-FIX denoised rsfMRI data (81) were used. Since the BF ROI is not included in the dense timeseries provided by HCP, these were regenerated using the updated subcortical parcellation to include the BF ROI voxels for further processing. Subsequent processing included ROI-constrained subcortical smoothing to match the cortical sampling density using the scripts provided by HCP (78), as well as additional signal filtering (i) based on the average WM and CSF timeseries using *ciftify* (82) and (ii) by applying a Wishart filter as proposed previously (83, 84) to selectively smooth unstructured noise more than the structured blood oxygen level-dependent signal. Average cortical ROI timeseries (concatenated across runs) were then extracted using the HCP-MMP 1.0 surface parcellation (35). Functional correlation maps were calculated by calculating the Pearson’s correlation coefficient for each voxel within the BF to each of the cortical parcels. The resulting correlation maps were reduced to a 2-dimensional *m*-by-*n* matrix, where *m* represent the voxels in the BF ROI (599 voxels) and *n* are the cortical targets (180 each hemisphere) with their corresponding functional correlation. This *m*-by-*n* connectivity matrix for all 173 subjects were averaged over subjects to calculate group-wise gradients.

### Gradient Calculation

Connectivity gradients were calculated using the BrainSpace toolbox (20). Group averaged connectivity matrices were used as input to the *GradientMaps* function, using the normalized angle kernel and diffusion map embedding approach. This nonlinear dimension reduction method transforms the connectivity matrix into a low-dimensional representation to construct connectivity gradients (85). BF voxels that are characterized by similar connectivity patterns will have a gradient value closer together, whereas voxels with litter or no similarity are farther apart. These gradients were then mapped back onto the BF voxel space to visualize transitions in functional and structural connectivity patterns.

In addition, gradient-weighted cortical maps were created by multiplying each row of the BF-cortical connectivity matrix with the corresponding gradient value of that BF voxel (45) (Supplemental Fig. S2). The distribution of cortical gradient-weighted values was then decomposed into seven functional networks (28) using the HCP-MMP 1.0 parcellation-based Yeo networks as defined in ref (28, 86). These networks include visual, somatomotor, dorsal attention, ventral attention, limbic, frontoparietal, and default mode.

### Weighted Residual map of BF

Weighted residual values for each of the BF voxels results were reconstructed by regressing the structural gradients against the functional gradients of BF and computing the residuals. First, residuals of all the pairs of selected gradients were calculated. For each pair of structure-function correlation, a combined weight was computed by adding the variance of corresponding structural and functional gradient components. This weighting was multiplied by the squared residual values and all 24 pairs were summed to produce the average weighted residual map of BF:

$$\bar{x}=\sum_{k=1}^{n}\left(w_{i,k}+w_{j,k}\right)x_{k}^{2}$$

where *k* is the structure-function pair, *w*_*i*_ is the explained variance of structural gradient and *w*_*j*_ is the explained variance of functional gradient, *x*_*k*_ is the residual values of *k* structure-function pair, and *n* is the number of structure-function pairs (i.e. 24).

### Geodesic Distance

Geodesic distance along the cortical surface was calculated using the geodesic library (https://github.com/the-virtual-brain/tvb-gdist) based on the algorithm that approximates the exact distance along the shortest path between two nodes (or vertices) on a triangulated surface mesh (87). An average BF seed node was created for the left and right hemispheres separately by (i) projecting the BF mask onto the *59k_fs_LR* white matter surface of the individual subjects using Connectome Workbench’s *volume-to-surface-mapping* function, (ii) averaging across all subjects to get a probability map, (iii) resampling to the *10k_fsavg* surface-space as suggested by the HCP study (https://wiki.humanconnectome.org) and (iv) then by thresholding at 0.5 to obtain a final binary BF seed on the cortical surface. A distance value was then assigned to each cortical vertex based on the minimum geodesic distance along the *10k_fsavg* pial surface to the BF seed node, hereby avoiding the medial wall. To match with the resolution of the cortical connectivity results, the geodesic distance map was parcellated using the HCP-MMP 1.0 atlas as implemented in the neuromaps toolbox (49), and rescaled to values between 0 and 1 (88).

### PET FEOBV maps

Positron emission tomography (PET) data were chosen to compare with our geodesic distance map (Fig. 3A left) and gradient-weighted cortical residual map (Fig. 2C top). These are [¹⁸F]fluoroethoxybenzovesamicol ([^18^F]FEOBV) imaging data targeting the vesicular acetylcholine transporter (VAChT) protein (51, 52). Each individual [^18^F]FEOBV PET image was intensity normalized to the subject’s supratentorial white matter uptake to create a parametric [^18^F]FEOBV PET image (53). The original PET atlases were transformed to *10k_fsavg* surface-space and parcellated to HCP-MMP 1.0 surface (35). The values of each cortical parcel encoding the relative concentration of cholinergic nerve terminals were rescaled (88) and visualized on an inflated surface (Fig. 4A). Spatial spin tests (48, 49) were used to statistically quantify the relationship between the geodesic distance map and cortical residual map. Additional FEOBV maps (89, 90) were obtained from the Neuromap toolbox (49) to examine the reproducibility of our results.

### Myelin Map

Individual T_1_-weighted divided by T_2_-weighted (T_1_w/T_2_w) as a proxy measure for intracortical myelin maps made available in the HCP minimally-preprocessed data (30), were averaged across subjects to create a group myelin map. This group myelin map was then transformed to the *10k_fsavg* surface space and parcellated using the HCP-MMP 1.0 atlas and values were rescaled for quantification of its relationship with cortical residual map.

### Statistical analyses

Permutation tests with surrogate maps (91) were used to compute statistical significance for the BF gradients and the distribution of residuals. BF gradient values for structural and functional connectivities were first rescaled between 0 and 1 and Euclidean distance was used to calculate the distance matrix among all voxels within the original BF ROI (88). Variograms were then permuted (N=1000) using the *SurrogateMaps* function implemented in the BrainSpace toolbox (20). Parameters were adjusted in the case of a suboptimal fit compared to the empirical data (pv=60, random_state=1234). The final variograms were used to build and compare mean and variation null probabilities between BF subregions.

Spin tests (48), as implemented in the neuromaps toolbox (49), were used to compare cortical maps based on N=10k permuted maps. All cortical maps were parcellated using the HCP-MMP 1.0 atlas (20) and values were rescaled between 0 and 1 (88). In addition to the spin tests, a bootstrapping analysis was performed to quantify the difference between structural and functional connectivity and their correlation with geodesic distance. Here, bootstrapping was applied 10k times (by randomly selecting sets of regions during each iteration) to build a null probability of correlation coefficients for statistical inference based on the empirical difference between the two modalities.

## Supplementary Material

Supplement 1
