# Supplementary material for: Multimodal gradients of human basal forebrain connectivity
Source: bioRxiv. 2023 May 26:2023.05.26.541324. Preprint. [Version 1] doi: 10.1101/2023.05.26.541324 (PMC10245994; doi:10.1101/2023.05.26.541324)
Supplement: Supplement 1 [file media-1.pdf]

Supplementary Information

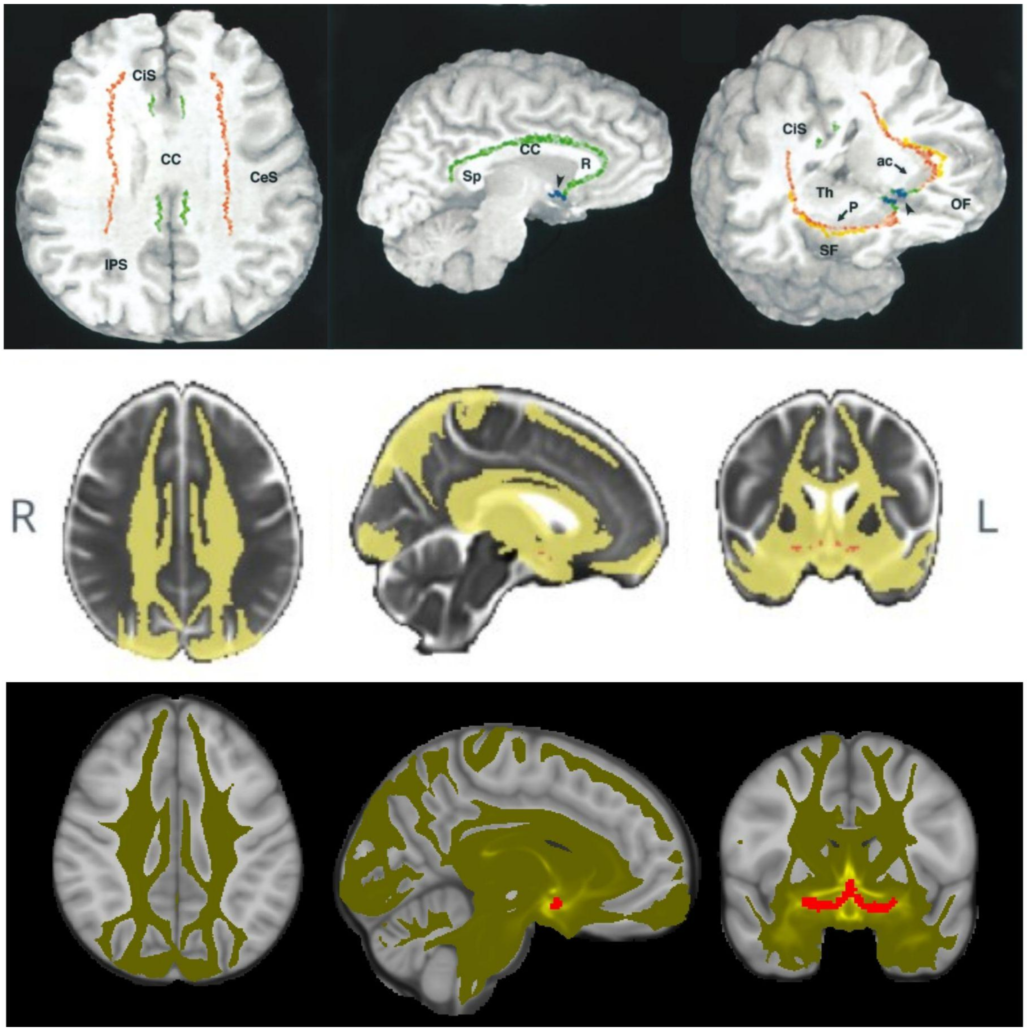

Fig. S1. Cholinergic pathways. The top row shows results from direct retrograde and anterograde labeling of cholinergic (ChAT+) fibers from the nucleus basalis of Meynert in human postmortem data (1). The basal forebrain nuclei are visible on the coronal oblique section. Two core cholinergic BF projections were identified: a medial cingulum pathway (green) and a lateral capsular pathway (red) with a perisylvian division (orange). The middle row shows *in vivo* diffusion MRI tractography of the nucleus basalis of Meynert in a large sample of adults (N=262) (2). Note that the medial and lateral tracts identified in vivo closely recapitulate the post-mortem tracing results. The bottom row shows the diffusion tractography results from our study. Our findings again show the strongest weighting of medial (cingulum) and lateral (capsular) pathways emanating from the basal forebrain.

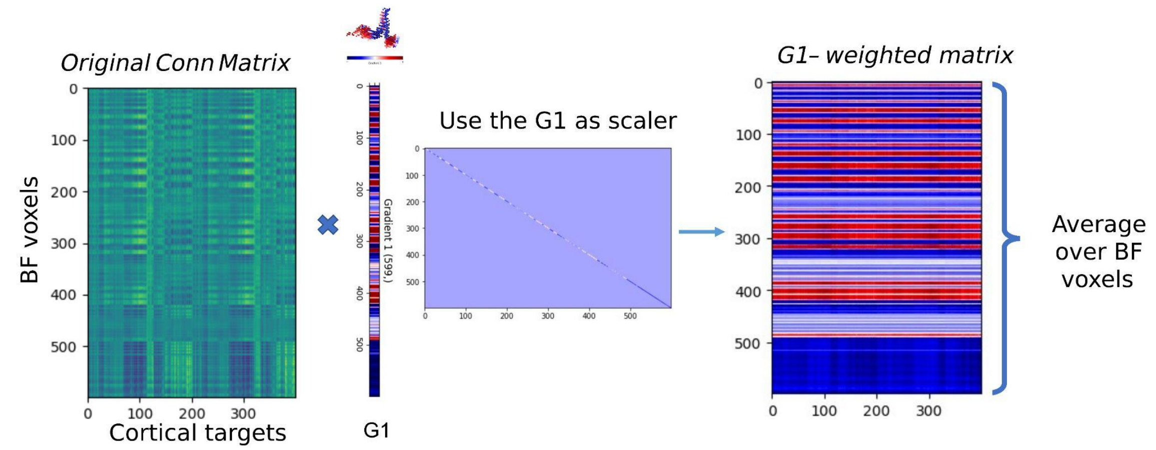

Fig. S2. Gradient-weighted cortical mapping method. Gradient-weighted cortical maps were created by first, multiplying each row of the initial connectivity matrix ($M_{\text{BF voxels}}$ x $N_{\text{cortical parcels}}$) in left with the corresponding gradient value of that BF voxel from gradient result (e.g. G1 here). This produce a G1-weighted connectivity matrix ($G_{\text{BF voxels}}$ x $N_{\text{Cortical parcels}}$), where all rows of the matrix (i.e. $G_{\text{BF voxels}}$) were averaged to produce a single cortical representation of the particular gradient.

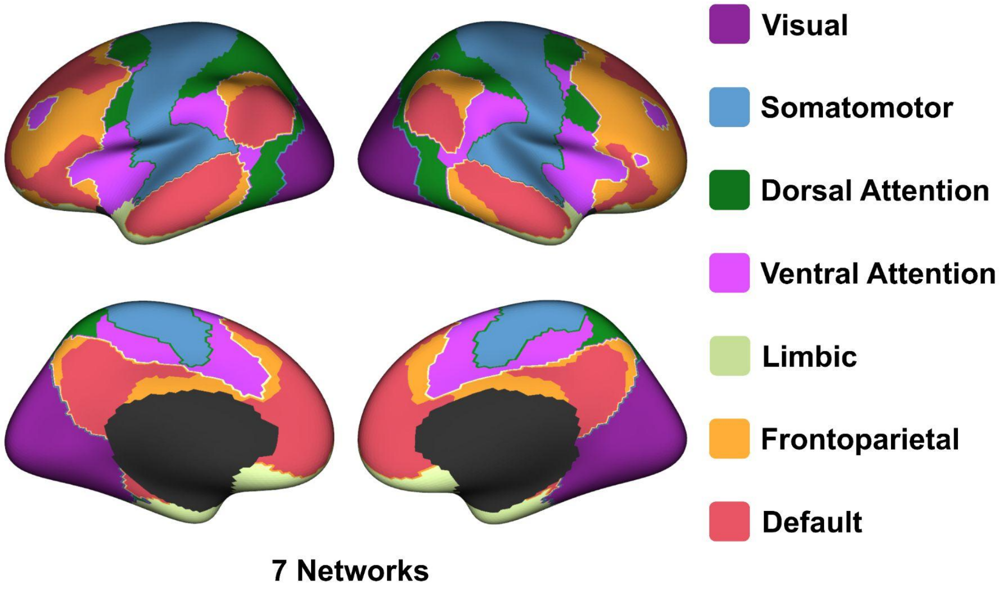

Fig. S3. A seven–network parcellation based on resting-state fMRI of the human cerebral cortex by ref (3, 4).

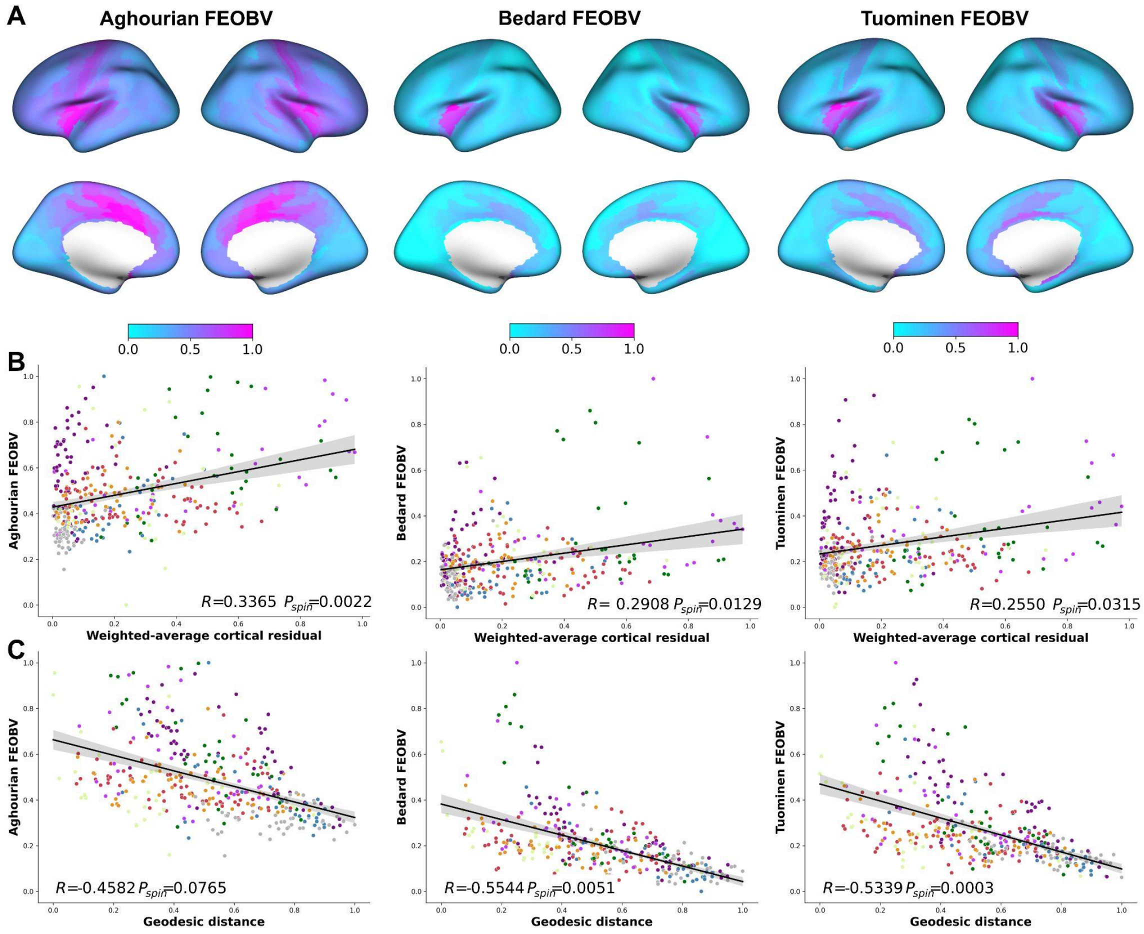

Fig. S4. Cortical cholinergic innervation in relation to cortical maps of weighted residuals and geodesic distance. Three publicly available FEOBV PET maps (5, 6) were obtained from the Neuromap toolbox (7)**;** they were parcellated, rescaled and projected on the cortical surface (A), pink indicating higher values while sky blue color indicating lower with the cortical BF label indicated by black spot. (B) Scatter plot against the weighted cortical residual map indicating positive correlation. Each point in the scatter plot represents cortical parcels based on the Glasser parcellation (8) and is color-coded by the 7 networks (3) identical to Fig. S2. Spin test using spatial null model (9) results are reported in the box corresponding to the scatter plots. (C) Scatter plot of the FEOBV PET maps against the geodesic distance showing negative relationship.

SI References

1. N. R. Selden, D. R. Gitelman, N. Salamon-Murayama, T. B. Parrish, M.-M. Mesulam, Trajectories of cholinergic pathways within the cerebral hemispheres of the human brain. *Brain* **121**, 2249–2257 (1998).

2. M. Nemy, *et al.*, Cholinergic white matter pathways make a stronger contribution to attention and memory in normal aging than cerebrovascular health and nucleus basalis of Meynert. *Neuroimage*, 116607 (2020).

3. B. T. T. Yeo, *et al.*, The organization of the human cerebral cortex estimated by intrinsic functional connectivity. *J. Neurophysiol.* **106**, 1125–1165 (2011).

4. L. Byrge, D. P. Kennedy, High-accuracy individual identification using a “thin slice” of the functional connectome. *Netw Neurosci* **3**, 363–383 (2019).

5. M. Aghourian, *et al.*, Quantification of brain cholinergic denervation in Alzheimer’s disease using PET imaging with [18F]-FEOBV. *Mol. Psychiatry* **22**, 1531–1538 (2017).

6. M.-A. Bedard, *et al.*, Brain cholinergic alterations in idiopathic REM sleep behaviour disorder: a PET imaging study with 18F-FEOBV. *Sleep Med.* **58**, 35–41 (2019).

7. R. D. Markello, *et al.*, neuromaps: structural and functional interpretation of brain maps. *Nat. Methods* (2022) https:/doi.org/10.1038/s41592-022-01625-w.

8. M. F. Glasser, *et al.*, A multi-modal parcellation of human cerebral cortex. *Nature Publishing Group* **536**, 171–178 (2016).

9. A. F. Alexander-Bloch, *et al.*, On testing for spatial correspondence between maps of human brain structure and function. *Neuroimage* **178**, 540–551 (2018).
